# Statistics of Infectious Diseases1Read before the Buffalo Academy of Medicine, Tuesday evening, September 12, 1893.

**Published:** 1893-11

**Authors:** Franklin C. Gram

**Affiliations:** Registrar of Vital Statistics, Department of Health, Buffalo, N. Y.


### STATISTICS OF INFECTIOUS DISEASES.[1]

By FRANKLIN C. GRAM, M. D.

Registrar of Vital Statistics, Department of Health, Buffalo, N. Y.

UNDER ordinary circumstances, statistics prove a dry subject, but when we examine those of infectious diseases our interest increases as we proceed.

I have compiled, in as brief a manner as possible, during the short period at my disposal, a summary of the statistics of all the principal infectious diseases recorded in the office of the Registrar of Vital Statistics, for this city, beginning with the year 1886—the first in which a complete detailed statement was published. Experience and acquired knowledge gradually improved the system of securing and compiling the necessary statistics in this department, so that today it stands as near the realization of perfection as any of the best standards adopted in this country.

I shall first present the table in its entirety :

| | 1886 | 1887 | 1888 | 1889 | 1890 | 1891 | 1892 | Total for Seven Years. | Per cent. of Deaths from Infectious Diseases. | Per cent. of Total Deaths. |
|---|---|---|---|---|---|---|---|---|---|---|
| Consumption | 426 | 456 | 460 | 440 | 498 | 549 | 546 | 3,375 | .335 | .09457 |
| Cholera Infantum | 344 | 436 | 393 | 314 | 347 | 381 | 376 | 2,591 | .257 | .07482 |
| Diphtheria | 122 | 183 | 174 | 130 | 111 | 165 | 177 | 1,062 | .105 | .03066 |
| Croup | 147 | 147 | 136 | 76 | 124 | 177 | 149 | 956 | .095 | .02760 |
| Typhoid Fever | 61 | 77 | 68 | 73 | 98 | 129 | 98 | 494 | .049 | .01484 |
| Scarlet Fever | 40 | 100 | 88 | 50 | 21 | 67 | 84 | 450 | .045 | .01299 |
| Cerebro-Spinal Meningitis | 70 | 62 | 47 | 58 | 89 | 73 | 49 | 448 | .045 | .01293 |
| Whooping Cough | 20 | 24 | 17 | 61 | 18 | 14 | 76 | 230 | .023 | .00664 |
| Measles | 5 | 60 | 35 | 5 | 56 | 47 | 18 | 226 | .022 | .00652 |
| Erysipelas | 13 | 13 | 18 | 10 | 14 | 17 | 26 | 111 | .011 | .00325 |
| Syphilis | 7 | 12 | 12 | 7 | 13 | 11 | 15 | 77 | .008 | .00222 |
| Small-Pox | .... | .... | 44 | 3 | .... | .... | .... | 47 | .005 | .00135 |
| Total | 1,255 | 1,570 | 1,492 | 1,227 | 1,389 | 1,630 | 1,614 | 10,067 | ...... | ...... |
| Total from all Causes | 3,869 | 4,688 | 4,929 | 4,328 | 5,117 | 6,001 | 5,697 | 34,629 | ...... | ...... |

1. Read before the Buffalo Academy of Medicine, Tuesday evening, September 12, 1893.

Let us now analyze the foregoing table, and make a few additions as we proceed.

Influenza is not mentioned therein, as I only found it in the summary for the year 1891, which stated that the remarkable number of fifty-five deaths were ascribed to this cause. Considering the prevailing fad at that time, physicians were either very successful in treating this disease, or they certified to the true cause of death, whenever the alleged "grip" robbed them of a patient. So common was it to call everything by that name, that you will pardon me for mentioning two cases, both of which were treated by old and successful practitioners, and with similar remedies. The first was that of a middle-aged lady, who had erysipelas of the face in an unmistakable degree. It was pronounced a case of "grip," and phenacetine was prescribed. The second case was that of a man who was suffering from an acute attack of prostatitis and orchitis. This was promptly designated as "grip," and he was given the same kind of treatment as the woman got for her face.

Of the total number of fifty-five, who were certified to as having succumbed to influenza, five were under one year, two between three and four years, five between twenty and thirty, one between thirty and forty, three between forty and fifty, ten between fifty and sixty, thirteen between sixty and seventy, and sixteen were seventy and over. There were twenty-four males, thirty-one females, and only one colored.

Small-pox, you will note, were only mentioned during the years 1888–89, when forty-seven deaths from that disease occurred in this city. During the same period, about 250 deaths occurred in this State from the same cause. The records at the Quarantine Hospital show that during the epidemic, lasting from January 24, 1882, to July 7, 1883, forty-nine cases of small-pox were treated at that institution, with only seven deaths, or about fourteen per cent.; while, during the last epidemic, 114 cases were treated at the Quarantine Hospital, from July 16, 1888, to October 12, 1889, with thirty-three deaths, or about twenty-nine per cent. There were 147 cases in all, with forty-seven deaths.

Considering the large number of cases of measles which occur, the mortality rate from this disease is much less, and more uniform than that of most of the infectious diseases. Its variability is seen by the foregoing table, where there is a difference of 1,200 per cent. in two consecutive years. In 1886, there were only five deaths from measles in this city, while the following year this number jumped to sixty; in 1888, it dropped to thirty-five, then came down to where it was in 1886, then jumped to fifty-six in 1890, and represented forty-seven and eighteen deaths, respectively, in the two succeeding years. Of those dying from measles at different periods, from ninety to 100 per cent. were below the age of five years.

Scarlet fever prevailed to the greatest extent in 1887, when a general increase in its mortality was noted throughout the State. It reached its minimum point in 1890, and continued at an unusually low rate throughout that year, when only twenty-one deaths, out of a total of 5,117, were ascribed to that cause in this city. It again began an upward tendency the following year, giving a total of eighty-four deaths last year, while the statistics for this year will doubtless exceed that point.

Diphtheria stands third on the list of infectious diseases, and omitting cholera infantum from this class, as is done by some authorities, it ranks second. It has constituted a considerable part of the mortality of this as well as other localities, being almost constantly present. Comparing diphtheria, croup, scarlet fever, whooping cough, and measles, we find that the ratio between diphtheria and scarlet fever is more proportionate in each year than that of the others. Whenever the ratio of one increases, the other does likewise. The greatest mortality from this disease, during the past seven years, was reported in 1887, when it reached 183 deaths out of a total of 4,688, and the smallest in 1890, when the number was lowered to 111. Of those dying, about ninety-five per cent. were under ten years of age. The average mortality for the seven years shows a rise of one-third in the mortality of October over that of September, from this cause, clearly indicating that the closing up of buildings and exclusion of fresh air from cellars are important factors in the causation of this disease. A study of the statistics in the various reports of the State Boards shows a much larger proportion of deaths from this disease in the cities than in the country.

Croup ranks next to diphtheria as regards mortality. I use the simple term "croup" because in the reports no distinction is made of the various kinds; and, right here, I beg permission to call the attention of physicians to the importance of giving all the information at their command when filling out death certificates.

The mortality from whooping cough is slightly in excess of that from measles, having been greatest in 1889 and 1892.

Cerebro-spinal meningitis and erysipelas show a greater uniformity in their death-rates than most of the other infectious diseases.

Cholera infantum, with all its terrors, does not claim as many victims as consumption. The former is found almost exclusively in the statistics of the hottest months of the year, but the latter we have always with us. My short experience in the Registrar's office happens to cover the cholera infantum period. A careful study of the death returns convinces me that the term "cholera infantum," as used by many practitioners, is about as indefinite as the term "heart disease," and in discussing the subject with some, I found that they made no distinction between gastric catarrh, dysentery, colitis, enteritis, entero-colitis, gastro-enteritis, and cholera infantum, but called any one of these by the latter name. This would indicate that the statistics on cholera infantum are misleading, caused by the fact that there is a diversity of opinion as to what really constitutes that disease.

Enteric, or typhoid, fever shows its greatest mortality between the ages of twenty to thirty, the next in rank being the ages of thirty to forty, while the period between fifteen to twenty comes third.

I added syphilis to this table, at the request of one of the gentlemen who is to discuss the subject tonight. Looking at the summary as here shown, and comparing it to the large number of cases of syphilis known to be extant, it would appear that this disease is not always stated as the cause of death when it ought to be. Taking the summary for last year, I find that out of a total of fifteen deaths five occurred in children under one year, three between the ages of twenty to thirty, four between the ages of thirty to forty, one between forty to fifty, one between fifty to sixty, and one between sixty to seventy years. There is very little variation between this and the tables for the previous years. But in this table I also find that no distinction is made between hereditary and acquired syphilis, so we are left to draw our own inferences by studying the ages. Perhaps one of the reasons for this is that if a young man, between the ages of twenty to thirty, would give a history of hereditary syphilis he might be suspected of not telling the truth as regards himself.

For the sake of comparison, the mortality of infectious diseases in the entire State, for the year 1890, is herewith presented. It is taken from the last published report of the State Board, and is as follows: Total deaths from all causes, 116,830; consumption 13,831; croup and diphtheria, 4,915; typhoid fever, 1,612; measles, 1,161; whooping cough, 1,156; scarlet fever, 913; cerebro-spinal fever, 476; erysipelas, 312; small-pox, 4.

I did not enter into any discussion of consumption because the statistics speak for themselves.

In conclusion, I desire to say that the true value of these statistics will depend entirely upon the accuracy preserved by the one who gathers the original information, namely, the practitioner.

